# Human Brain Responses and Functional Brain Networks Across the Lifespan

**DOI:** 10.3390/brainsci15040402

**Published:** 2025-04-16

**Authors:** Birgit Mathes, Canan Başar-Eroǧlu

**Affiliations:** 1 Faculty of Human and Health Sciences, University of Bremen, 28359 Bremen, Germany; 2 Faculty of Arts and Science, Izmir University of Economics, 35330 Izmir, Turkey; canan.basar@izmirekonomi.edu.tr

## 1. Introduction

Measuring brain responses in real time using electrophysiology enriches our understanding of changes in behavior and cognitive function across the lifespan [1]. Life-long adaptations of human brain networks allow responding to life-period-specific challenges. This enables learning and improvement of new abilities during development and compensational processes during aging [2,3,4]. Research investigating human brain responses across the lifespan further allows us to understand risks of emerging mental health or neurodegenerative disorders [5,6,7]. Recent studies increasingly mirror how brain development and function are influenced by social experiences and socioeconomic status, i.e., the social and economic position of a person or group within society, typically measured by income, education, and occupation [8,9,10,11].

Adaptations in human brain networks through life can be characterized by spontaneous and event-related activity. Characterizing oscillatory EEG measures specifically enables determining age-related changes in the temporal coordination of multiple neural activation patterns and their integration within functional neural networks [12]. Both spontaneous and event-related measures allow the implementation of similar or even identical routines in data acquisition and analysis, thus supporting a focus on age-related changes. Abilities of life-long importance, including attention and response inhibition, can be investigated with well-established paradigms, such as the oddball or Go/NoGo task across age [13,14,15,16,17].

The aim of this Special Issue was to bring together a broad range of EEG and other neuroscientific studies to better understand the mechanisms and functions of brain changes through the lifespan. In the following, you will find a short description of all contributions in the order of age groups, ranging from 4 months to 86 years.

## 2. Overview of Published Articles

1. Neural responses related to social influences and cognitive abilities in infants and toddlers

Infancy is characterized by fast learning of basic and life-long important abilities as well as increased neuroplasticity, meaning the adaptation of brain structure and function due to learning experiences [18,19]. Living circumstances may thus have a lasting effect on brain maturation. Its impact on cognition may root in infancy and increase over the life course. Peykarjou et al. (2024) demonstrate that fast visual characterization of living and nonliving objects is already developed in 4-month-old children. Tarullo et al. (2024) and Wienke and Mathes (2024) investigate how early living circumstances may affect early neural development.

- Tarullo et al. (2024) undertook a challenging longitudinal resting-state EEG study in rural South Africa. They aimed to gain better insights into the determinants of early brain maturation at 7, 17, and 36 months of age from children growing up with restricted resources. Their results indicated that larger head size at birth predicted smaller gamma and larger theta power, particularly at 7 months of age. This finding, when looking at high-SES literature, counterintuitively indicates delayed maturation in children with a presumably better fetal environment. Tarullo and colleagues argue that high-SES children, in comparison to children experiencing more environmental prenatal challenges, may experience a prolonged time window of increased neural plasticity, an important mechanism for life-long learning and cognitive performance. Taken together, their studies underline the importance of reflecting social context in developmental neuroscience.
- In a comprehensive study, Peykarjou et al. (2024) demonstrate that 4-month-old infants already elicit a brain response indicative of categorizing visual stimuli into “living” and “non-living”. Their elegant task design enables Peykarjou et al. (2024) to study fast categorization abilities and their neural correlates similarly in infants of 4, 7, and 11 months, as well as in 6–7-year-old children and young adults. In all groups, categorization responses emerged at posterior-occipital and frontal leads and were driven mostly by global Gestalts and to a lesser degree by low-level visual features. The early emergence and similar, though not identical, neural categorization response across age points towards the life-long importance to distinct living, and potentially interacting, from nonliving objects.
- The EEG study of Wienke and Mathes (2024) examined how the brain responses of infants aged 6 to 14 months are influenced by their families’ socioeconomic background. During a passive auditory oddball paradigm, the results showed an immature N2-P3a complex, with the N2 amplitude being more positive for deviants than for standards. This effect was more pronounced in infants from families with lower parental education and with a recent migration history, especially when burdens coincide. This suggests that early neural development is affected by socioeconomic and cultural challenges. The observed deviations may have an impact on attention and learning processes early on.

2. Neural responses related to social influences and cognitive abilities during childhood

This Special Issue includes three reviews, all approaching changing neural networks during childhood with a different focus. Karakaş (2024) summarizes EEG studies on attentional processes and emphasizes studying brain oscillations. Accordingly, Ünsal et al. (2024) provide an update on functions of brain oscillations during neurodevelopment. The review of Schneider et al. (2024) ties into the discussion of how the environment and socioeconomic position of a child correlates with brain function.

- The review of Karakaş (2024) spans between the roots and underlying concepts of EEG research, how these relate to up-to-date neurodevelopmental studies on attention in children and directions for future research. Event-related potentials (ERPs) are commonly used to investigate early and late stages of attentional processing in children. ERP latencies indicate slower processing speed in children than adults, while the overall shape, topography, and amplitude of ERPs indicate qualitative differences and, thus, the developmental reorganization of neural networks between childhood and adulthood. Investigating brain oscillation may allow a better understanding of spatial and temporal integration within parallel processing neural networks during childhood. Developmental changes in spontaneous oscillations are discussed as the building blocks of event-related brain oscillations (EROs) and the superposition of event-related oscillations as the building blocks of ERPs; thus, Karakaş (2024) stresses the necessity to better understand their inter-relation in neurodevelopmental research. She further demonstrates the importance of understanding clinical applications of attentional processing deficits originating in childhood.
- Schneider et al. (2024) provide a comprehensive and future-directed review illustrating environmental risk factors on brain maturation and cognitive functioning. Low maternal education and low family income are often correlated but may affect the brain of the maturing child via distinct pathways. Less diverse linguistic input, often a result of low educational attainment, affects the development of the language processing system. Low income may lead to increased everyday stress, which affects brain development globally and may have detrimental effects for the child’s cognitive control and executive functions. The authors further propose that cultural context, including family size, parental beliefs, and living area, need to be considered more thoroughly in future research.
- Ünsal et al. (2024) review the state of knowledge regarding event-related brain oscillations between infancy and childhood. Following a thorough literature search, they summarize functional correlates as well as age-related changes in event-related delta, theta, alpha, beta, gamma, and mu oscillations across 86 studies. Theta oscillations have been researched most often so far, as they play a role in perception, attention, and memory. The reviewed studies demonstrate that in children, similar to adults, oscillatory responses of all frequency bands are important for cognitive functions. The authors emphasize the fruitful approach of understanding brain development through an age-related view on brain oscillations. They conclude that future studies should strongly focus on the interplay between brain responses in different frequency bands and further integrate advanced neuroimaging and EEG techniques.

3. Neural responses related to language abilities during adolescence

- Behboudi et al. (2023) illustrate the maturation of ongoing gamma oscillations in early adolescents (8 to 15 years) elicited during sentence processing. Broadly increased ongoing gamma activation was observed in younger participants. Older participants showed a more localized activation pattern at left central and posterior regions and smaller amplitude values than younger participants. In concert with a previous study of their group [20], the authors argue that theta and gamma networks elicited during sentence processing still mature at the onset of adolescence. Their work may be taken as an example of how oscillatory neural networks become better defined during adolescence, which may specifically reduce the need for word-by-word integration for sentence comprehension.

4. Structural and Functional Brain Networks during young adulthood

Young adulthood seems to be a period of relative stability in brain function and underlying structure. Subtle age-related changes may, however, be found. Fehr et al. (2024) and Rizzo et al. (2024) analyze brain oscillations during young adulthood. Salisbury et al. (2024) use MRI data to investigate if the onset of a mental illness during young adulthood accelerates early aging of the brain.

- Fehr, T. et al. (2024) present a thorough signal- and source-based analysis of age-related changes in resting-state EEG during the third decade of life. EEG power remained relatively stable across age in all major oscillatory frequency bands (delta, theta, alpha, beta, and gamma). Network activation pattern, as indicated by the succession of activations in modeled sources of the EEG signal, changed across age. The findings comprised different and broadly distributed sources for different frequency bands. The authors conclude that age-related changes in the third decade are subtle and affect how information is processed (as indicated by network activation pattern) rather than source strength (as indicated by power).
- Rizzo et al. (2024) explore neural oscillatory dynamics underlying reduced one-sided pain perception utilizing the mirror-induced feedback illusion. Watching tapping one’s right finger in a mirror while the left finger is unmoved and hidden elicits an illusion of the left finger moving. During the illusion, painful versus painless electrical stimulation of the hidden finger induced increased desynchronization of alpha activity. This trend was reversed in two control conditions, indicating that the illusion affected cortical activation. The authors argue that the illusion changed internal body perception and allocation of attentional resources to the painful stimulus during both anticipation and experience of pain.
- Salisbury et al. (2024) present structural imaging data from healthy young adults and patients approximately 27 days (median) after their first clinical contact due to a suspected psychotic disorder. Previous studies reported signs of early aging in patients with established schizophrenia and progressive gray matter loss when transitioning into a first psychosis [21,22]. Power analysis of the authors make it, however, unlikely that early aging of the brain is found early during the course of a psychotic illness. They report older predicted brain age in patients with poorer emotional expressivity, i.e., in relation to the extent of negative symptoms, as well as with lower premorbid IQ, i.e., in relation to life-long and pre-illness acquisition of knowledge.

5. Changes in neural responses and cognition across childhood to late adulthood

Two cross-sectional studies investigate changes in brain function covering a broad age range. Barry et al. (2024) looks at pre-stimulus EEG and post-stimulus ERPs during response inhibition and execution in children, young and old adults. Yener et al. (2024) utilize a visual oddball paradigm to investigate the effects of age and gender on event-related delta and theta oscillations during young, middle, and late adulthood.

- Barry et al. (2024) thoroughly explore the links between pre-stimulus EEG, post-stimulus ERPs, and performance during Go and NoGo processing in children (8–12 years) as well as young (18–24 years) and older adults (59–74 years). Children showed the lowest and young adults showed the highest performance. In accordance, young adults elicited the simplest, indicating the most efficient, pattern of links between EEG/ERP and performance measures. The links between EEG/ERPs and performance were more complex in children and older adults. This result may be indicative of less effective modulation of attentional and vigilance states and of reduced cognitive control. Brain maturation may lead to better distinction between Go and NoGo processing.
- Yener et al. (2024) utilize a classical visual oddball paradigm and analyze amplitude differences in delta and theta oscillations across adulthood (18–86 years) and gender. Their rationale was that development of Alzheimer’s disease is twice as likely in women than men and slow oscillations elicited by targets relate to abnormalities in cognitive performance. Their results indicate that decline of theta amplitudes with age is steeper in women, leading to increased theta amplitudes in women compared to men until middle adulthood. These results may relate to early signs of dementia being more likely in women than in men. The authors call for gender-stratified and gender-adjusted analysis in future studies on aging to overcome women’s under-representation in research and better adjustment of treatment strategies according to gender.

## 3. Conclusions

Contributions in this Special Issue covered age groups ranging from infancy to old age. Studies focused on age factors in healthy participants and patients with psychotic symptoms. Three contributions focused on environmental influences on brain function and overcoming under-representation of people with socioeconomic challenges in research. Paradigms included resting EEG, passive stimulation, and active behavioral paradigms, with a focus on attentional states, language comprehension, and cognitive control. Multifold analytical methods were applied, looking at time frequency components of the oscillatory dynamics of the brain, source localization, and ERP measures as well as the link between EEG/ERP and behavioral performance. Thus, EEG measures provide important and various opportunities to measure life-long changes in brain function, its relation to cognition, risk factors, and the course of mental illnesses.

One of the presented studies included longitudinal data and another MRI data. Future direction of lifespan research would profit from more longitudinal studies, ideally covering longer age ranges and a combination of methods to integrate information from brain structure and neural dynamics. Mobile EEG studies would allow for brain dynamics to be investigated in real-life situations. We further stressed that increasing diversity in research and understanding how life experiences and living circumstances relate to brain neuroplasticity remain important to reach better representability and to improve prevention and support strategies and medical treatment. These considerations call for collaboration between research institutions and research disciplines, data sharing, and long-term studies that allow for comprehensive recruitment and multiple testing. Future research therefore faces both challenges and opportunities to gain comprehensive insights into brain dynamics and their real-life implications across the lifespan.
